# Processing and storage methods affect oral and gut microbiome composition

**DOI:** 10.3389/fmicb.2023.1253570

**Published:** 2023-10-03

**Authors:** Dorothy K. Superdock, Wei Zhang, Angela C. Poole

**Affiliations:** ^1^Division of Nutritional Sciences, Cornell University, Ithaca, NY, United States; ^2^School of Integrative Plant Science, Cornell University, Ithaca, NY, United States

**Keywords:** saliva, dental, gut, microbiome, frozen, lyophilized, freeze dried, liquid nitrogen

## Abstract

In microbiome studies, fecal and oral samples are stored and processed in different ways, which could affect the observed microbiome composition. In this study, we compared storage and processing methods applied to samples prior to DNA extraction to determine how each affected microbial community diversity as assessed by 16S rRNA gene sequencing. We collected dental swabs, saliva, and fecal samples from 10 individuals, with three technical replicates per condition. We assessed four methods of storing and processing fecal samples prior to DNA extraction. We also compared different fractions of thawed saliva and dental samples to fresh samples. We found that lyophilized fecal samples, fresh whole saliva samples, and the supernatant fraction of thawed dental samples had the highest levels of alpha diversity. The supernatant fraction of thawed saliva samples had the second highest evenness compared to fresh saliva samples. Then, we investigated the differences in observed community composition at the domain and phylum levels and identified the amplicon sequence variants (ASVs) that significantly differed in relative abundance between the conditions. Lyophilized fecal samples had a greater prevalence of Archaea as well as a greater ratio of Firmicutes to Bacteroidetes compared to the other conditions. Our results provide practical considerations not only for the selection of storage and processing methods but also for comparing results across studies. Differences in processing and storage methods could be a confounding factor influencing the presence, absence, or differential abundance of microbes reported in conflicting studies.

## 1. Introduction

Assessment of oral and gut microbial communities has become prevalent in clinical studies as therapeutics targeting the microbiome are increasingly explored (Wade, 2013; Lynch and Pedersen, 2016). Methods used during the workflow while generating microbial genetic sequencing data can vary widely between studies, including sample collection method, storage, and DNA extraction, which can all influence observed microbial composition (Song et al., 2016; Hugerth and Andersson, 2017; Kim et al., 2017; Vandeputte et al., 2017; Teng et al., 2018; Fiedorová et al., 2019; Cunningham et al., 2020; Marotz et al., 2021). If these methods affect the detection of the microbes that mediate the disease or treatment being studied, research groups may report conflicting results. It is, therefore, important to consider the impact of differences in storage and processing methods before making comparisons across studies. The objective of this current study was to compare the microbiome compositions observed in oral and gut samples that were stored and processed in different ways.

**Figure 1:**
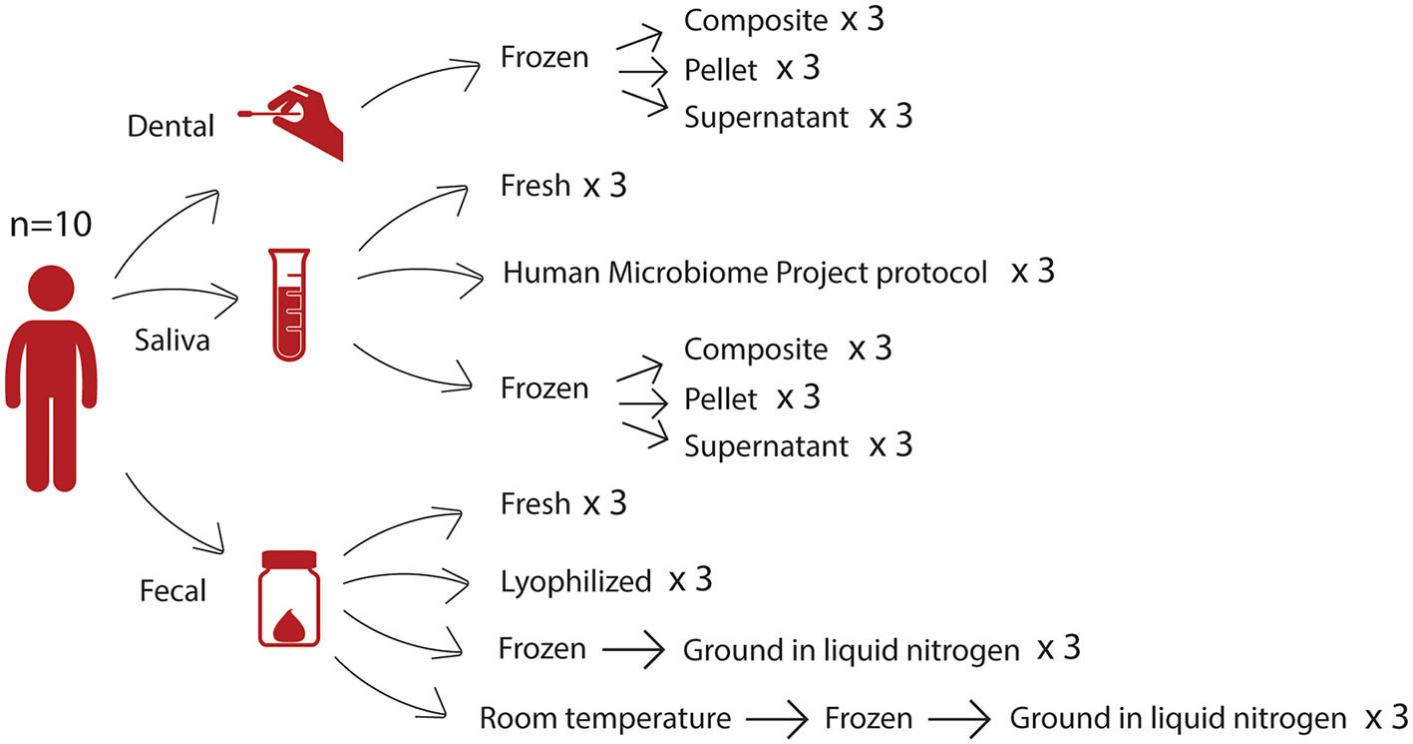
Study design. From 10 donors, we collected three different sample types—dental swabs, saliva samples, and fecal samples—which were then subjected to several storage and processing methods. For each sample, each condition was tested in triplicate.

We collected samples from 10 individuals, and each sample was subjected to several different storage and processing methods (Figure 1), hereafter referred to as conditions, in triplicate, prior to DNA extraction and 16S rRNA gene sequencing. For fecal samples, we investigated how lyophilization differed from other conditions, as this long-term preservation method eliminates the need for cold storage. Due to the removal of water, lyophilization reduces sample mass and allows for indefinite storage at room temperature without further microbial growth. We compared lyophilized fecal samples to three other conditions: (1) fresh fecal samples (from which DNA was extracted on the day of collection without first being frozen), (2) fecal samples frozen at −80°C then subsequently ground in liquid nitrogen (LN2), and (3) fecal samples that were left out at room temperature prior to freezing at −80°C and then ground in liquid nitrogen (LN2post72hr). This final condition was intended to mimic studies in which fecal samples are shipped to investigators, who then freeze the samples until further processing. In this study, we used a 72-h incubation time to simulate 3 days at room temperature. The LN2 and LN2post72hr samples were ground in liquid nitrogen before being added to the extraction plate because the lyophilized samples were ground after lyophilization, and we sought to exclude grinding as a confounding factor.

We also examined differences between thawed fractions of oral samples, specifically saliva samples and dental swabs. Saliva samples are commonly collected to determine the overall oral microbiota composition. For studies involving caries or periodontal disease, dental swabs can be collected to enhance the detection of bacteria that form biofilms on teeth in a less invasive manner than collecting subgingival plaque (Lu et al., 2022; Uyghurturk et al., 2022; Selway et al., 2023). These sample types are commonly frozen and then thawed before processing. Upon thawing, there is often precipitation of proteins, such as mucins, present in the samples (Schneyer, 1956). Researchers may make different decisions at the input step of a DNA extraction protocol regarding whether to use centrifugation to separate the sample into a pellet and supernatant or vortexing to homogenize the sample. We compared the three different fractions of thawed saliva and dental samples (pellet, supernatant, or composite) to determine which part of the sample should be targeted for DNA extraction when it is not possible to use a fresh sample. The supernatant and pellet fractions resulted from the centrifugation of the thawed sample, and the composite fraction was a vortexed mixture of the whole thawed sample prior to the centrifugation step used to separate the supernatant and pellet fractions. In addition, for saliva samples, we evaluated the Human Microbiome Project (HMP) Phase 1 “Initial Processing of Saliva” protocol, which was developed by the HMP as part of a common set of guidelines and protocols (https://www.hmpdacc.org/), as well as a fresh condition where samples were subject to DNA extraction on the day of collection without first being frozen.

## 2. Materials and methods

### 2.1. Human subjects

We collected fecal, saliva, and dental swabs from each of 10 healthy participants (*n* = 6 women, *n* = 4 men) between 25 and 50 years of age (Cornell University IRB protocol #1106002281). For saliva samples and dental swabs, participants were instructed to refrain from brushing their teeth for at least 6 h and refrain from consuming any food or beverages including water for at least 30 min before sample collection. Samples were treated using several different processing and storage methods as shown in Figure 1 and described below. Hereafter, we refer to these different combinations of processing and storage methods as conditions.

### 2.2. Saliva samples

Each participant secreted ~5 ml of saliva into a 50-ml conical tube. Each saliva sample was vortexed, and (1) 500–750 μl was pipetted into a PowerSoil Bead Tube (MO BIO PowerSoil DNA Isolation Kit, QIAGEN Cat #12888) for DNA extraction within 2 h of collection (fresh), (2) 1 ml was pipetted into a microcentrifuge tube and was centrifuged at 2,600 × *g* for 15 min at room temperature, then 750 μl of supernatant was added to 750 μl of PowerSoil Bead Buffer and frozen at −80°C prior to DNA extraction (HMP), and (3) 1 ml was pipetted into another microcentrifuge tube and was frozen without centrifugation. After thawing frozen aliquots, samples contained varying amounts of precipitate and were vortexed so that an aliquot containing both liquid and precipitate was removed with a wide orifice pipette tip to represent the composite fraction. Then samples were centrifuged at 1,500 × *g* at 4°C for 15 min to separate a pellet from the supernatant fraction.

### 2.3. Dental swabs

Participants were asked to simulate brushing the buccal side of their teeth, top and bottom rows, for 30 s with nylon swabs (Epicenter, cat # QEC89100). Six dental swabs were collected per participant. Dental swabs were placed in bead solution from the MO BIO PowerSoil kit for 5–10 min, swirled, and vortexed briefly on the lowest setting to facilitate the transfer of dental swab material into the bead solution. A measure of 100–200 μl of bead solution containing dental swab material was then combined for each participant, split into triplicate, and then frozen at −80°C within 90 min of collection. Upon thawing, each of the three samples was first mixed to obtain the composite aliquot, and then centrifuged at 1,500 × *g* at 4°C for 15 min to separate a pellet from the supernatant fraction.

### 2.4. Stool samples

Participants collected their own stool samples using a commode specimen collection kit (Thermo Fisher, cat # 02-544-208). After receipt by the lab on the day of collection, each stool sample was mechanically homogenized in a zip-top freezer bag using a rolling pin and transferred into 50 ml conical tubes containing no preservative or buffer for further processing downstream using four different methods. The samples were (1) processed fresh, (2) lyophilized (freeze-dried), (3) frozen at −80°C for >2 weeks, then ground in liquid nitrogen (LN2), or (4) stored at room temperature for 72 h, frozen at −80°C for >2 weeks, then finally ground in liquid nitrogen (LN2post72hr). The lyophilized samples were ground by placing steel rods in conical tubes containing the sample and then rotating the conical tubes on a rock tumbler (Poole et al., 2019). All samples across all conditions, except those intentionally left out at room temperature for 72 h, were at room temperature for the same amount of time during aliquoting prior to downstream processing.

### 2.5. 16S rRNA library generation and sequencing

Microbial DNA was extracted from all gut and oral samples using the MO BIO PowerSoil-htp Soil DNA Isolation Kit (MO BIO Laboratories, cat # 12955-4) according to the manufacturer's protocol, except instead of vortexing, the samples were placed in a BioSpec 1001 Mini-Beadbeater-96 for 2 min after the addition of Solution C1. For the amplification of the V4 region of the 16S rRNA gene, we used 10–50 ng of sample DNA in duplicate 50 μl PCR reactions with 5 PRIME HotMasterMix and 0.1 μM forward (515F) and reverse (806R) primers using the PCR program previously described (Caporaso et al., 2011) but with 25 cycles. We purified amplicons using the Mag-Bind E-Z Pure Kit (Omega Bio-tek, cat # M1380) and quantified them with Invitrogen Quant-iT PicoGreen dsDNA Reagent. A total of 100 ng of amplicons from each sample were pooled and sequenced on an Illumina MiSeq instrument using 2 × 250 bp paired-end sequencing. We used QIIME2 version 2022.2 (Bolyen et al., 2019) to perform microbiome bioinformatics. We imported sequences as EMPPairedEndSequences. We demultiplexed and quality-filtered raw sequence data using q2-demux. The number of raw FASTQ sequencing reads for each sample included in our analyses can be found in Supplementary Table 1. We denoised using DADA2 (Callahan et al., 2016) via q2-dada2, with no trimming, but truncated sequence reads at the first base where the bottom 25th percentile of reads dropped below a *q*-score of 30, as observed in the visualizations generated by q2-demux. We created a phylogenetic tree using q2-fragment-insertion using the sepp-refs-gg-13-8.qza reference database and calculated diversity metrics using q2-diversity after sequences from the samples were rarefied. Only alpha and beta diversity analyses used rarefied sequence data: 31,915 sequences per gut sample and 19,360 sequences per oral sample. For analyses where saliva samples and dental samples were analyzed separately, diversity metrics were calculated separately (for microbial diversity analyses, we excluded the HMP condition for saliva samples to avoid unbalanced groups). To assign taxonomy to ASVs, we used the classify-sklearn naïve Bayes taxonomy classifier via the q2-feature-classifier plugin (Bokulich et al., 2018) against Greengenes 13_8 99% OTU reference sequences (McDonald et al., 2012). Although samples were all processed in triplicate, some of the sample replicates failed at some point during the library preparation workflow and failed to produce sufficient yields for sequencing. Additionally, only 12 out of the 30 samples in the HMP condition reached the sequencing step of the library preparation workflow, while the rest failed to produce sufficient yields for sequencing. In total, for the rest of the saliva samples, there were 28 fresh, 30 composite, 30 pellet, and 25 supernatant samples included in the analyses. For fecal samples, there were 30 lyophilized, 30 fresh, 30 LN2, and 30 LN2post72hr samples included in the analyses. For dental swabs, there were 23 composite, 28 pellet, and 28 supernatant samples included in the analyses.

### 2.6. Statistical analysis

We performed all statistical analyses in RStudio using R version 4.2.1 (R Core Team, 2022). To determine how microbiome composition differs at the phylum and domain levels, we used taxa exported from taxa bar plots generated by QIIME2 and used the lme4 R package to fit linear mixed models with condition as a fixed effect and sample donor as a random effect, e.g., lmer (Archaea Relative Abundance ~ Condition + (1|SubjectID)), separately for each sample type. For fecal samples, we tested whether the Firmicutes:Bacteroidetes ratio was different depending on condition by fitting the following model: FBratio ~ Condition + (1|SubjectID). To compare microbial communities between samples, we calculated unweighted (taking into account the presence/absence of microbes only) and weighted (also accounting for relative abundances of microbes present) UniFrac distances as measures of beta diversity using the QIIME2 diversity plugin core-metrics-phylogenetic. We then visualized these distances using principal coordinate analysis (PCoA) and performed PERMANOVA analysis to compare within-group to between-group beta diversity distances using adonis2 of the vegan R package (Oksanen et al., 2022) and pairwise.adonis2 (Martinez Arbizu, 2017), stratifying by donor whenever donor was not used as a fixed effect in the models, i.e., using the parameters “permutations=” (adonis2) or “strata=” (pairwise.adonis2) in the model formulas. We checked the PERMANOVA assumption of the homogeneity of variance in dispersions using the betadisper function in the vegan package and found the assumption to be met in all oral models and all but one fecal model. This assumption was not met in the fecal model that tested differences between-condition groups using weighted UniFrac distances. However, this was acceptable based on the fact that condition groups were balanced (Anderson and Walsh, 2013). We used set.seed (123456) for all of our beta-diversity analyses. For our pairwise comparisons using pairwise.adonis2, we manually adjusted for multiple comparisons using the *p*.adjust function, using the Benjamini–Hochberg procedure. Principal coordinate analysis (PCoA) plots were generated using vegan (Oksanen et al., 2022). We performed alpha diversity analyses using the lme4 R package to fit linear mixed models using each alpha diversity metric—Faith's Phylogenetic Diversity (Faith's PD, phylogenetic diversity), Pielou's Evenness (evenness), or Observed ASVs—as the response variable, condition as a fixed effect, and subject as a random effect, e.g., using the following equation for each alpha diversity metric: Alpha Diversity ~ Condition + (1|SubjectID). We performed this analysis separately for each sample type. For all linear mixed models, we performed *post-hoc* pairwise comparisons employing the emmeans R package using the Tukey method for *p*-value adjustment. For the alpha diversity correlation analysis, we averaged Faith's PD for saliva samples and dental samples within each subject and performed Pearson's correlation. We used MaAsLin2 (Mallick et al., 2021) to evaluate the effect of conditions on the relative abundances of microbes. We used default settings except setting normalization to none as we used relative abundance tables as input that were already normalized by total sum scaling. For each of our MaAsLin2 models, we included condition or fraction as a fixed effect and subject as a random effect. We included all sample replicates per condition by merging ASVs from technical replicates.

## 3. Results

### 3.1. Microbiome composition differs at the phylum and domain levels between fecal, dental, and saliva conditions

**Figure 2:**
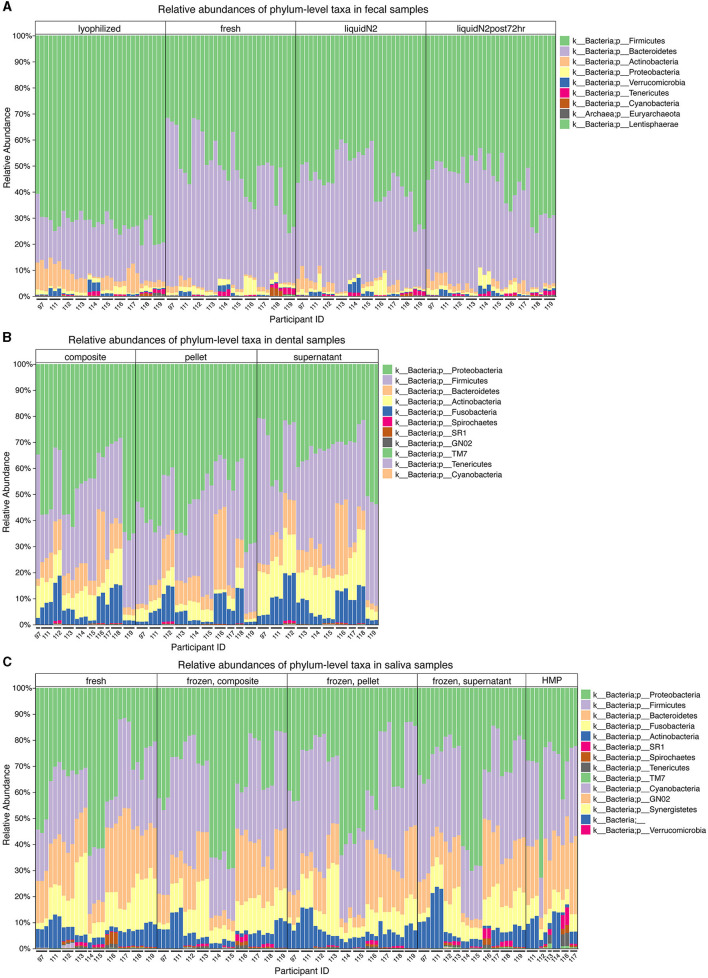
Microbial taxa differ at the phylum level for different fecal and oral sample storage and processing methods. **(A)** Relative abundances of the most prevalent microbial taxa in fecal samples categorized by condition (lyophilized, fresh, LN2, and LN2post72hr). **(B)** Relative abundances of the most prevalent microbial taxa in dental samples categorized by thawed fraction (composite, pellet, and supernatant). **(C)** Relative abundances of the most prevalent microbial taxa in saliva samples categorized by condition (fresh, composite, pellet, supernatant, and HMP). **(A–C)** All legends list taxa in order from most to least abundant. **(A–C)** Taxa that were not visible due to low relative abundances are not listed in the figure key. Microbial taxa grouped by donor can be found in Supplementary Figure 1.

Effects of the different conditions were observed at the phylum level. Notably, in the fecal samples, there were differences in the proportions of the top two dominant phyla in the gut, Firmicutes and Bacteroidetes (Figure 2A). Compared to the other three conditions (fresh, LN2, and LN2post72hr), lyophilization was associated with a greater than two-fold increase in the ratio of Firmicutes to Bacteroidetes (*p* < 0.0001). Lyophilized samples also had a higher relative abundance of Actinobacteria compared to all other conditions (*p* < 0.0001). LN2post72hr samples had a higher relative abundance of Actinobacteria compared to fresh samples as well (*p* = 0.03). Fresh samples had a higher relative abundance of Tenericutes compared to lyophilized (*p* = 0.01) and LN2 samples (*p* = 0.03). Fresh samples also had a higher relative abundance of Proteobacteria compared to lyophilized samples (*p* = 0.049). Additionally, there were differences observed at the highest taxonomic rank (domain) level in fecal samples, as detectable by 16S rRNA sequencing. Archaea were only detected in 36 out of 120 total fecal samples and only in five of the 10 donors. In two of these five donors, Archaea was detected in only a single replicate sample: six out of 133,346 ASVs and 18 out of 102,727 ASVs. Within the samples from the remaining three donors, lyophilization was associated with an increased relative abundance of Archaea compared to fresh (*p* = 0.009), LN2 (*p* = 0.004), and LN2post72hr (*p* = 0.04).

Relative abundances of phyla observed in the oral samples are shown in Figures 2B, C, with the top two dominant phyla being Proteobacteria and Firmicutes for both saliva and dental samples. Dental supernatant contained a higher relative abundance of Firmicutes compared to composite and pellet, but a lower relative abundance of Proteobacteria compared to composite and pellet (*p* < 0.0001 for all). Dental pellet also contained a higher relative abundance of Proteobacteria compared to composite (*p* < 0.0001). The relative abundance of Firmicutes, but not Proteobacteria, was significantly affected by condition in saliva samples. Fresh saliva contained a lower relative abundance of Firmicutes than composite (*p* < 0.0001), HMP (*p* = 0.002), pellet (*p* < 0.0001), and supernatant (*p* < 0.0001). Pellet contained a higher relative abundance of Firmicutes compared to composite (*p* = 0.03) and HMP (*p* = 0.01).

### 3.2. Lyophilization strongly affects the beta diversity of fecal samples

**Figure 3:**
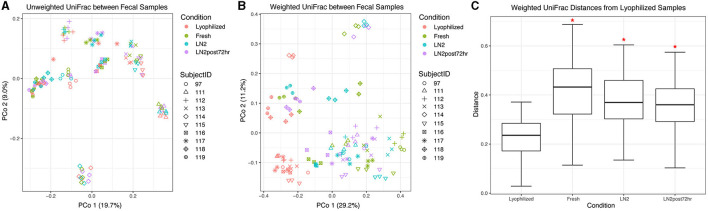
Beta diversity of fecal samples. Principal coordinate analysis plot of **(A)** unweighted and **(B)** weighted UniFrac distances between fecal samples, colored by condition. Shapes represent different subjects. **(C)** Box plot representing pairwise weighted UniFrac distances between each condition and lyophilized fecal samples. Red asterisks (*) indicate significantly different distances from lyophilized samples as determined by PERMANOVA without stratification by donor.

We found that donor was the primary driver of fecal sample clustering using unweighted UniFrac distances, explaining 77% of the variation in distances between samples (*R*^2^ = 0.77, *F* = 39.98, *p* = 0.001; Figure 3A). However, the condition still explained a statistically significant amount of the variation (*R*^2^ = 0.029, *F* = 1.14, *p* = 0.005; Figure 3A). Samples more strongly clustered by condition based on weighted UniFrac distances compared to unweighted UniFrac distances (Figure 3B) in a principal coordinate ordination. Visualizing weighted distances, lyophilized samples appeared to cluster away from the other conditions, and, correspondingly, all conditions had significantly different community compositions (*R*^2^ = 0.27, *F* = 14.58, *p* = 0.005). Donor identity explained less of the variation between weighted distances than between unweighted distances (*R*^2^ = 0.59, *F* = 17.48, *p* = 0.001; Figure 3B). For both unweighted and weighted UniFrac distances, we then performed pairwise PERMANOVA analyses, stratified by donor, to test which conditions were significantly different from one another within each donor. We found that between-condition distances were significantly greater than within-condition distances for all conditions (*p* < 0.05), except for LN2 vs. LN2post72hr. However, when using weighted UniFrac distances, lyophilization had a strong effect such that the lyophilized sample cluster was significantly different from all the other conditions even without stratification by donor (*p* = 0.002 for all pairwise comparisons with lyophilized; Figures 3B, C).

### 3.3. Condition affects the beta diversity of dental samples

**Figure 4:**
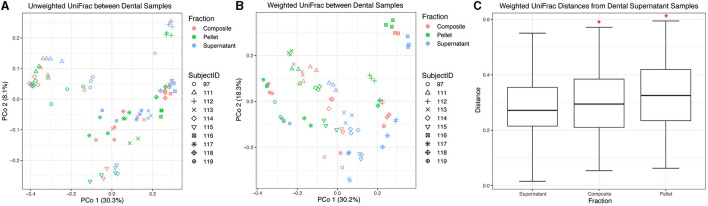
Beta diversity of dental samples. Principal coordinate analysis plot of **(A)** unweighted and **(B)** weighted UniFrac distances between dental samples, colored by fraction. Shapes represent different subjects. **(C)** Box plots represent pairwise weighted UniFrac distances between each dental fraction and dental supernatant. Red asterisks (*) indicate significantly different distances from dental supernatant samples as determined by PERMANOVA without stratification by donor.

Next, we compared the beta diversity of dental samples obtained from thawed fractions. Using unweighted UniFrac distances, we found that donor identity explained most of the variation between samples (*R*^2^ = 0.73, *F* = 21.19, *p* = 0.001), with visually apparent clustering by donor (Figure 4A). Overall, the fraction had a small yet statistically significant effect (*R*^2^ = 0.02, *F* = 0.70, *p* = 0.005). Using weighted UniFrac distances, donor identity explained more of the variation between samples (*R*^2^ = 0.83, *F* = 37.46, *p* = 0.001) as compared to when using unweighted UniFrac distances, while fraction also explained more of the variation observed (*R*^2^ = 0.14, *F* = 5.93, *p* = 0.005) compared to unweighted distances (Figure 4B). We then performed a PERMANOVA stratified by donor using weighted UniFrac distances and found that all dental fractions were significantly different from one another (*p* = 0.001 for all pairwise comparisons). However, when using weighted UniFrac distances and considering all samples collectively without stratifying by donor, we found that only supernatant samples were significantly different from pellet (*p* = 0.003) and composite (*p* = 0.0135), while pellet and composite distances did not differ (*p* = 0.1520; Figure 4C).

### 3.4. Condition affects the beta diversity of saliva samples

**Figure 5:**
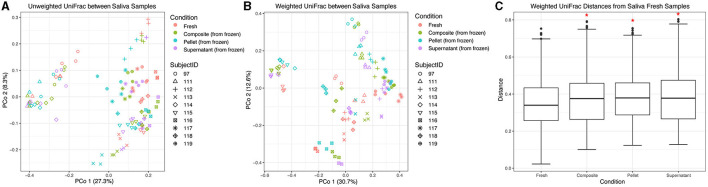
Beta diversity of saliva samples. Principal coordinate analysis plot of **(A)** unweighted and **(B)** weighted UniFrac distances between saliva samples, colored by condition. Shapes represent different subjects. **(C)** Box plots represent pairwise weighted UniFrac distances between samples in each condition and fresh saliva samples. Red asterisks (*) indicate significantly different distances from fresh saliva samples as determined by PERMANOVA without stratification by donor.

We also compared beta diversity between different fractions of thawed saliva and fresh saliva samples. The samples from the HMP processing method were excluded from this analysis due to fewer replicates with adequate sequencing depth, which would have resulted in unbalanced groups in the statistical models. Using unweighted UniFrac distances, similar to our dental sample results, we found that donor identity explained most of the variation between saliva samples (*R*^2^ = 0.73, *F* = 30.47, *p* = 0.001), and condition (which included fresh samples vs. composite, pellet, and supernatant fractions) was a minor but still statistically significant contributor (*R*^2^ = 0.078, *F* = 3.09, *p* = 0.005; Figure 5A). We also found that using weighted UniFrac distances increased the contribution of donor identity (*R*^2^ = 0.83, *F* = 54.16, *p* = 0.001) and decreased the contribution of condition (*R*^2^ = 0.068, *F* = 2.64, *p* = 0.005; Figure 5B) to the observed variation. Despite the minor contribution of condition relative to donor in explaining microbial community variation in saliva samples, when stratifying by donor using both weighted and unweighted UniFrac distances, we found that between-condition distances were significantly greater than within-condition distances for all pairwise comparisons (*p* = 0.001 for all weighted distances and *p* = 0.0012 for all unweighted distances except unweighted composite vs. supernatant, which was *p* = 0.048). Thus, despite the strong influence of donor identity, condition significantly affected microbial community differences between samples from the same donor. Without stratifying by donor, using both weighted and unweighted UniFrac distances, we found that only fresh samples were significantly different from the other conditions (*p* < 0.05; weighted UniFrac shown in Figure 5C).

### 3.5. Alpha diversity is highest in lyophilized fecal samples, dental supernatant, and fresh saliva samples

**Figure 6:**
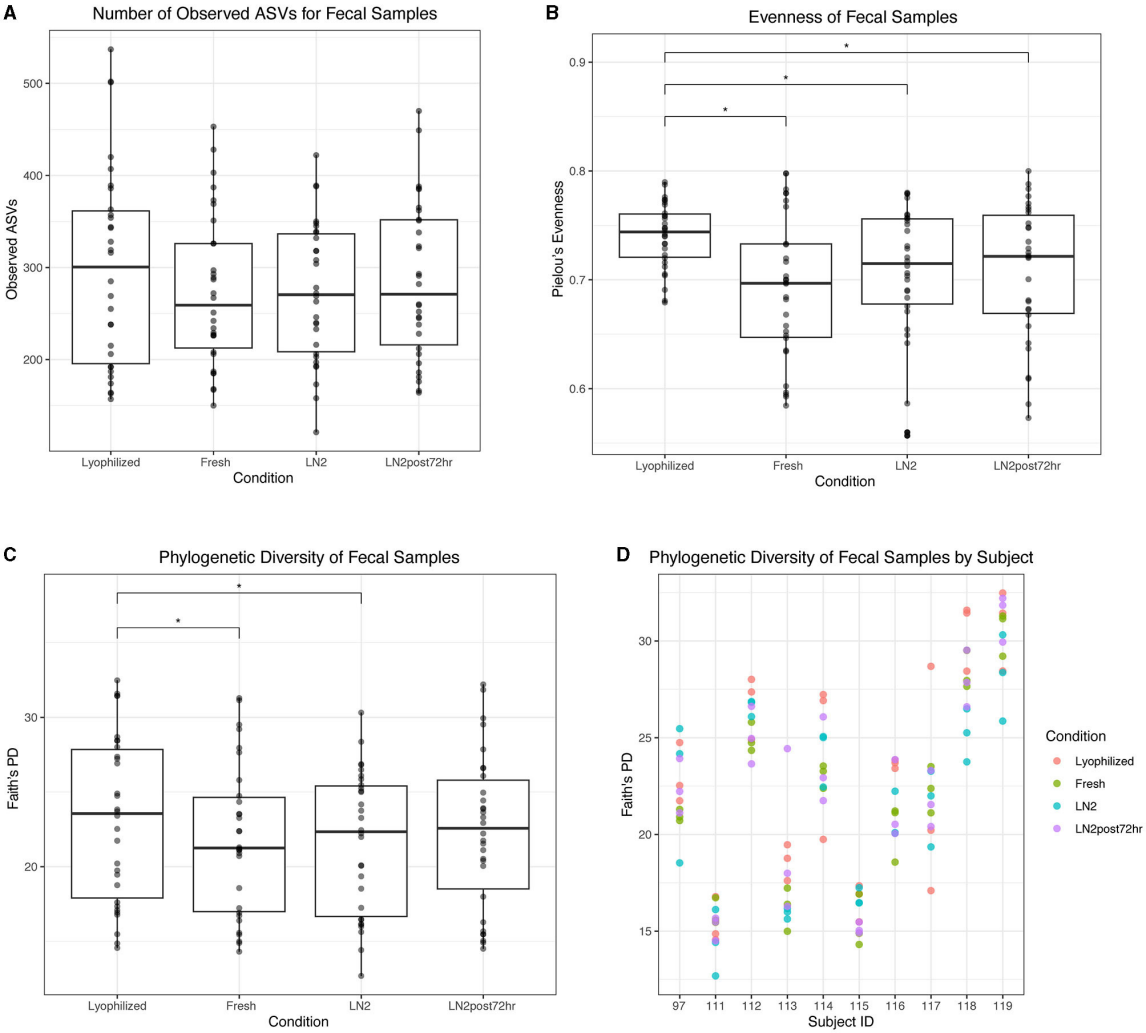
Alpha diversity of fecal samples. **(A)** Observed ASVs in fecal samples by condition. **(B)** Evenness of fecal samples by condition. **(C)** Phylogenetic diversity as determined by Faith's PD of fecal samples by condition. Statistically significant (*p* < 0.05) differences between groups as determined by pairwise comparisons are denoted by an asterisk (*) in **(A–C)**. **(D)** Phylogenetic diversity in individual subjects, colored by condition.

In fecal samples, lyophilized samples had the highest alpha diversity overall. Despite no significant differences in the number of observed ASVs based on condition (Figure 6A), we found that lyophilized samples had significantly greater evenness than fresh (*p* < 0.0001), LN2 (*p* = 0.0005), and LN2post72hr samples (*p* = 0.0007; Figure 6B), and significantly greater Faith's PD than fresh (*p* = 0.03) and LN2 samples (*p* = 0.01; Figure 6C). Faith's PD by individual donors is shown in Figure 6D. Interestingly, the LN2post72hr samples did not exhibit a greater range of standard deviation in Faith's PD between replicates for each donor (SD = 4.0) than the lyophilized samples (SD = 5.8) even though the LN2post72hr samples were stored at room temperature for 3 days with no added preservative.

**Figure 7:**
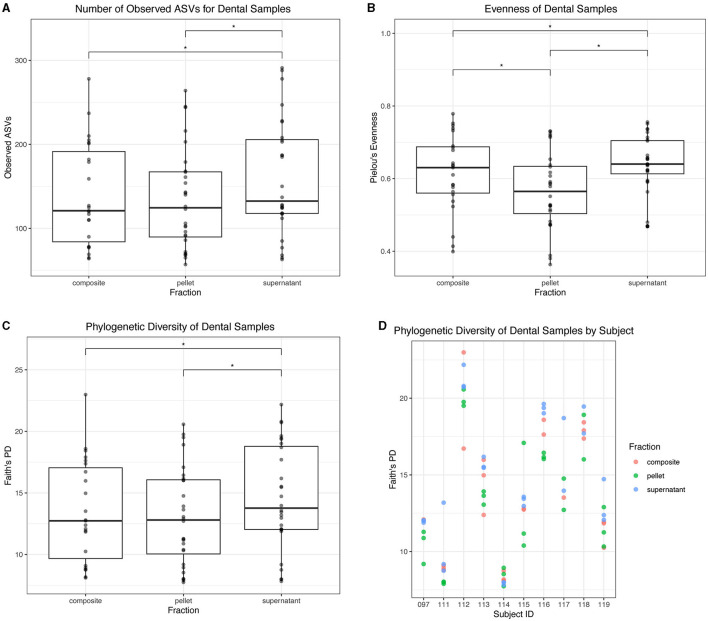
Alpha diversity of dental samples. **(A)** Observed ASVs in dental samples by fraction. **(B)** Evenness of dental samples by fraction. **(C)** Phylogenetic diversity as determined by Faith's PD of dental samples by fraction. Statistically significant (*p* < 0.05) differences between fractions are denoted by an asterisk (*) in **(A–C)**. **(D)** Phylogenetic diversity by individual subject, colored by dental fraction.

For dental samples, we found that the supernatant fraction had the highest alpha diversity. Supernatant had a greater number of observed ASVs than both composite (*p* = 0.0004) and pellet (*p* < 0.0001) fractions (Figure 7A). All fractions differed in evenness: supernatant had greater evenness than composite (*p* = 0.0001) and pellet (*p* < 0.0001), and composite had greater evenness than pellet (*p* < 0.0001; Figure 7B). Finally, supernatant had a greater Faith's PD than both composite (*p* = 0.02) and pellet (*p* = 0.0001; Figure 7C). Faith's PD in donor replicates is shown in Figure 7D.

**Figure 8:**
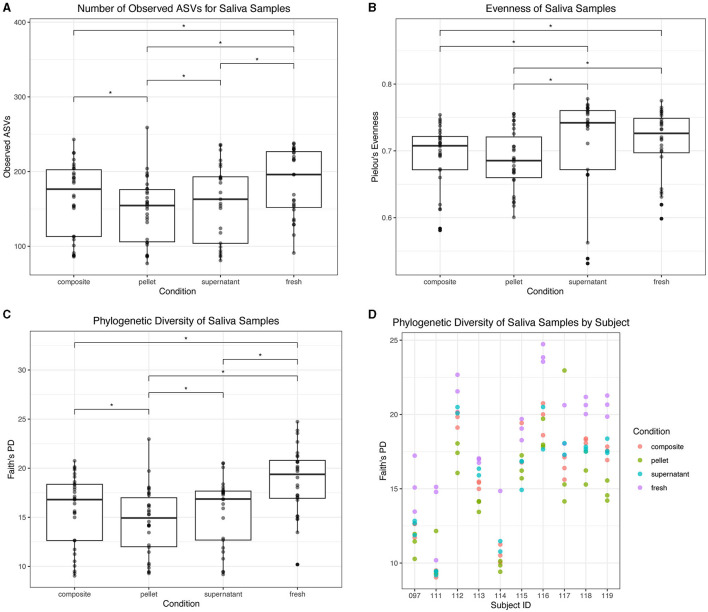
Alpha diversity of saliva samples. **(A)** Observed ASVs in saliva samples by condition. **(B)** Evenness in saliva samples by condition. **(C)** Phylogenetic diversity as determined by Faith's PD in saliva samples by condition. Statistically significant (*p* < 0.05) differences are denoted by an asterisk (*) in **(A–C)**. **(D)** Phylogenetic diversity by individual subject for each condition.

For saliva samples, we found that fresh saliva had the highest alpha diversity. Fresh saliva samples had a higher number of observed ASVs than all thawed fractions (*p* < 0.05; Figure 8A). Composite and supernatant both had a greater number of observed ASVs than pellet (*p* < 0.05; Figure 8A). Fresh saliva had greater evenness than pellet (*p* = 0.003) and composite (*p* = 0.02), and supernatant had greater evenness than pellet (*p* = 0.0003) and composite as well (*p* = 0.002; Figure 8B). However, there were no significant differences in evenness between fresh and supernatant or composite and pellet (*p* > 0.05; Figure 8B). Fresh saliva samples had the greatest Faith's PD compared to all frozen fractions (*p* < 0.0001; Figure 8C). While supernatant and composite were not significantly different from each other, they both had significantly greater Faith's PD than pellet (*p* = 0.01). Faith's PD in donor replicates for each saliva sample type is found in Figure 8D. Additionally, we found that the average Faith's PD across saliva samples and dental samples was correlated in donors (*r* = 0.88, *p* = 0.0009).

### 3.6. Differential abundance analysis of highest diversity samples

In addition to differences in diversity metrics, we identified microbes at the ASV level that distinguished the samples in the conditions with the highest alpha diversity from the others. We chose to compare the highest diversity condition with the others to identify the maximum number of ASVs that could be different between conditions. Thus, for fecal samples, we compared fresh, LN2, and LN2post72hr samples to lyophilized samples. After FDR-adjusting *p*-values to correct for multiple comparisons, there were 230 ASVs with relative abundances that were significantly different from lyophilized in total (92 ASVs differed between fresh and lyophilized, 61 ASVs differed between LN2 and lyophilized, and 77 ASVs differed between LN2post72hr and lyophilized; adjusted *p, q* < 0.05; Supplementary Table 2). The ASV that increased in relative abundance in both fresh and LN2post72hr compared to lyophilized was assigned to *Prevotella copri*. The ASV that was most increased in relative abundance in LN2 compared to lyophilized was assigned to *Bacteroides plebeius*.

For saliva samples, we compared composite, pellet, and supernatant to fresh samples. There were 145 ASVs that significantly differed from fresh in total (54 differed between pellet and fresh, 43 differed between composite and fresh, and 48 differed between supernatant and fresh; *q* < 0.05; Supplementary Table 2). The two ASVs that were most increased in relative abundance in pellet and composite compared to fresh were both assigned to *Veillonella dispar*, and the ASV that was most increased in relative abundance in supernatant compared to fresh was assigned to *Selenomonas*. For saliva samples, we also compared composite and pellet to supernatant, and we found that 21 ASVs were significantly different between composite and supernatant, and 70 ASVs were significantly different between pellet and supernatant (*q* < 0.05). The same ASV that was most increased in relative abundance in both composite and pellet compared to supernatant was assigned to *Prevotella*.

For dental samples, we compared composite and pellet to supernatant, our reference condition, as it had the highest alpha diversity among the sample fractions. There were 3 ASVs that were significantly different between supernatant and composite, and 33 ASVs that were significantly different between supernatant and pellet (*q* < 0.05; Supplementary Table 2). Out of these significant results, the ASV that was most increased in relative abundance in composite compared to supernatant was assigned to Pasteurellaceae. The ASV that was most abundant in pellet compared to supernatant was an ASV assigned to *Actinobacillus* (in the Pasteurellaceae family). All statistically significant results are presented in Supplementary Table 2.

A summary of our above findings can be found in Table 1.

**Table 1:** Summary of findings.

**Sample type**	**Condition with highest phylogenetic diversity**	**Significantly different ASVs**
Fecal	Lyophilized	230 ASVs different between lyophilized and the other three conditions (fresh, LN2, and LN2post72hr) • 92 ASVs different between fresh and lyophilized • 61 ASVs different between LN2 and lyophilized • 77 ASVs different between LN2post72hr and lyophilized
Dental	Supernatant	36 ASVs different between supernatant and the other two conditions (composite and pellet) • 3 ASVs different between composite and supernatant • 33 ASVs different between pellet and supernatant
Saliva	Fresh	145 ASVs different between fresh and the other three conditions (pellet, composite, and supernatant) • 54 ASVs different between pellet and fresh • 43 ASVs different between composite and fresh • 48 ASVs different between supernatant and fresh

This table includes a summary of our alpha diversity and differential abundance results for each sample type. In the last column, we compare the number of ASVs that differ between the condition that had the highest phylogenetic diversity and the other conditions.

## 4. Discussion

Our findings indicate that comparing microbial composition results across studies should be done with care when the storage or processing methods differ. We found that different conditions significantly affected the observed community composition in dental swabs, saliva, and fecal samples.

There are several possible mechanisms that could affect 16S rRNA gene abundance during storage and processing. For example, freezing fecal samples may affect microbial DNA differently depending on whether microbes are Gram-positive or Gram-negative and therefore differentially affect downstream PCR amplification (Bahl et al., 2012; Fouhy et al., 2015; Metzler-Zebeli et al., 2016).

Our most notable result was that lyophilized fecal samples had microbiomes that were most distinct from the other conditions. Based on our results, caution should be exercised when using lyophilization because not only did lyophilization result in higher alpha diversity than the other conditions, but it also resulted in a significantly greater proportion of Firmicutes to Bacteroidetes than samples in all three other conditions. Freezing has also previously been found to increase the Firmicutes to Bacteroidetes ratio (Bahl et al., 2012; Fouhy et al., 2015), and lyophilization could enhance this effect through a similar mechanism; since most Firmicutes are Gram-positive, their DNA may preferentially survive the dehydration process compared to DNA from Gram-negative Bacteroidetes. Although the Firmicutes to Bacteroidetes ratio has been posited as a marker of obesity (Ley et al., 2005; Mathur and Barlow, 2015; Koliada et al., 2017), this association is not consistent (Finucane et al., 2014; Sze and Schloss, 2016; Johnson et al., 2017; Magne et al., 2020). Our observation that a technical factor can affect this ratio further supports caution in the use of the Firmicutes to Bacteroidetes ratio as a biomarker.

Lyophilized fecal samples also had a significantly greater relative abundance of Archaea compared to samples in the other conditions, therefore indicating the ability of this processing method to increase the detection of microbes in this domain. Of note, having higher amounts of methanogenic Archaea in the gut has been correlated with obesity (Zhang et al., 2009; Basseri et al., 2012; Mathur and Barlow, 2015). It has also been reportedly unclear as to whether the predominant archaeal species, *Methanobrevibacter smithii*, is only present in a certain percentage of the population or whether a technical factor has hindered its detection (Gaci et al., 2014).

We also found that lyophilized fecal samples had less *P. copri* compared to fresh and LN2post72hr samples. Previous studies suggest *P. copri* as an important marker of various health-related conditions and outcomes. In Kovatcheva-Datchary et al. (2015), individuals with gut microbiomes enriched with *P. copri* had improved glucose metabolism following the consumption of dietary fiber-enriched bread, and gavaging mice on a high-fiber diet with *P. copri* resulted in improved glucose tolerance. Another study showed that mice treated with *P. copri* had lower glucose levels compared to the control group (Verbrugghe et al., 2021). Increased *P. copri* has also been suggested to play a role in rheumatoid arthritis (Scher et al., 2013; Jiang et al., 2022). In light of these examples, it is imperative to take storage and processing methods into account when comparing results between gut microbiome studies. Finally, for fecal samples, our beta diversity analysis revealed that condition better explains the variation between samples when accounting for microbial abundance (weighted UniFrac) rather than just microbial presence or absence, which suggests that the effect of condition is more prominent when giving less weight to low abundance ASVs.

Although distinctions between donors were generally maintained between conditions for oral samples, we detected some ASVs that were significantly enriched depending on the condition. Furthermore, supernatant had the highest alpha diversity out of the three thawed fractions from dental samples. *Selenomonas* was found to be the ASV that increased in relative abundance in thawed saliva supernatant compared to fresh. This genus includes species such as *Selenomonas noxia*, which has been associated with periodontitis disease (Tanner et al., 1998; Torresyap et al., 2003; Cruz et al., 2015). Fresh saliva had higher alpha diversity than any of the frozen fractions. If fresh saliva is considered to be the condition containing the most accurate representation of the true microbial community, we recommend using thawed composite if using fresh samples that have never been frozen is impossible because thawed composite has the least amount of ASVs differing from fresh. We acknowledge that it is rarely feasible, especially for high-throughput studies, to conduct DNA extractions immediately after receipt of a sample in the lab, particularly as samples are often not collected all at once. It is important to note that in this study the centrifugation speed that separated supernatant and pellet was relatively low (1,500 × *g* at 4°C for 15 min), and some protocols might recommend much higher centrifugation speeds to form a pellet in order to maximize DNA yield, in which case the supernatant may not contain as much DNA.

There is no “one size fits all” recommendation. The observation of higher alpha diversity for some conditions is not inherently concerning and could be indicative of an observed microbiome that is closest to the true composition of the sample *in vivo*. However, we cannot assume that the observed composition with the highest alpha diversity is more accurate. For example, when comparing fresh stool to lyophilized stool, it may be that this condition allows for enhanced detection of a subset of microbes, preferentially increasing their relative abundance. Thus, these differences in composition could be a drawback when lyophilized samples are compared with samples processed using other methods in a meta-analysis. However, lyophilization could be useful if Methanogens or Actinobacteria are the microbes of interest.

In conclusion, multiple metrics of microbiome composition were affected by storage and processing methods for both gut and oral microbiome samples, with some methods increasing observed microbial diversity over others and enriching a subset of ASVs that have been associated with various aspects of host health. Therefore, it is important to exercise caution when selecting a processing method for one's study as well as when comparing results across studies that use different processing methods.

## Data availability statement

The data presented in this study are deposited in the National Center for Biotechnology Information Sequence Read Archive (NCBI SRA) repository under BioProject PRJNA984696.

## Ethics statement

The studies involving humans were approved by Cornell University Institutional Review Board for Human Participant Research. The studies were conducted in accordance with the local legislation and institutional requirements. The participants provided their written informed consent to participate in this study.

## Author contributions

DS: Data curation, Formal analysis, Investigation, Methodology, Visualization, Writing—original draft, Writing—review and editing. WZ: Methodology, Writing—review and editing. AP: Conceptualization, Investigation, Methodology, Project administration, Supervision, Writing—review and editing, Writing—original draft.
